# Easy-handling semi-floating TiO_2_-based aerogel for solar photocatalytic water depollution

**DOI:** 10.1007/s11356-022-23772-5

**Published:** 2022-10-26

**Authors:** Sana Nouacer, Ridha Djellabi

**Affiliations:** 1 Laboratory of Water Treatment and Valorization of Industrial Wastes, Chemistry Department, Faculty of Sciences, Badji-Mokhtar University, BP12 2300 Annaba, Algeria; 2 École Nationale Supérieure Des Mines Et MétallurgieENSMM, Ex CEFOS Chaiba, BP 233 RP Annaba, W129 Sidi Amar, Algeria; 3 Department of Chemical Engineering, Universitat Rovira I Virgili, 43007 Tarragona, Spain

**Keywords:** Semi-floating photocatalyst, TiO_2_ aerogel, Solar photocatalysis, Cr(VI) photoreduction, Dye photooxidation

## Abstract

One of the capital issues of photocatalytic technology is how to use photocatalytic materials in real world conditions. Suspension photocatalysts are the most effective, while the handling and recovery of nanoparticles are very challenging and costly. Herein, we report the design of semi-floating aerogel TiO_2-_based photocatalyst for the oxidation of dyes and photoreduction of Cr(VI). TiO_2_ aerogel-based photocatalyst was fabricated through in situ polymerization using borax, poly(vinyl alcohol) and polyvinylidene in the presence of H_2_O_2_ as a catalyst. Cubic TiO_2_ aerogel of few centimetres was designed for the photocatalytic tests under solar light irradiation. TiO_2_ aerogel showed a good photoactivity against the oxidation of three types of dyes and Cr(VI) photoreduction. In terms of dyes, the kinetics of methylene blue oxidation was the fastest as compared to rhodamine B and methyl red, while, a total reduction of Cr(VI) at 10 ppm was obtained within 30 min after the addition of tartaric acid as hole scavenger. TiO_2_ aerogel can be easily recovered, washed and recycled. TiO_2_ aerogel can move freely from the top to the deep solution. The semi-floating property could be an advantage to enhance the mass transfer along with bulk solution, as compared to totally floating-based photocatalysts.

## Introduction

Water purification is one of the capital challenges of nowadays’ society due to the huge pollution resulted from intensive industrial, domestic and agricultural activities (Chaudhry and Malik 2017). As a consequence, alternative technologies are under investigation towards the fast and sustainable purification of wastewaters (Adeola and Forbes 2021, Yadav et al. 2022). New recommendations from the environmental community have been raised to develop alternative approaches for water purification by respecting several factors including the excellent efficiency, less use of chemical, sustainability of the system and the low cost. Photocatalysis technology is one of the emerging raised technologies over the last few decades. Basically, it is based on the photoexcitation of a photocatalyst with light irradiation having an energy higher or equal to the band gap of the photocatalyst (Chong et al. 2010, Wang et al. 2022). This excitation results in the in situ generation of oxidative reactive oxygen species (ROSs) which are able to oxidize unselectively most of organic pollutants. However, the photocatalytic oxidation kinetics depends on many factors which include the nature of the organic pollutant, the surface interaction between the pollutant and photocatalyst, the efficiency of the photocatalyst to produce ROSs, the potential of pollutant against the valence/conduction band of the photocatalyst and so on (Zhang et al. 2012, Ohtani 2014). Up to date, a lot of research studies have been reported on the use of photocatalysis towards the oxidation and reduction of organic and inorganic pollutants, by emphasizing the enhancement of the photocatalytic kinetics via the development of novel photocatalytic materials (Di Paola et al. 2012, Djellabi et al. 2022b) or/and photoreactors design (Marinho et al. 2017b, Alalm et al. 2021). In terms of materials design, several approaches have been developed to enhance the photocatalytic rate under solar light through doping (Zaleska 2008), photosensitizing (Robert 2007) and so on. On top of the visible light absorption, the current issues of photocatalytic technology are associated with other factors such as the generation of toxic by-products, the complications regarding how to use the photocatalyst and the photoreactor, low photocatalytic rates…etc., as discussed recently by Djellabi et al. (Djellabi et al. 2021a). To ensure the highest mass transfer and photocatalytic rate, the use of photocatalyst as suspension is the best option to get the highest contact between the photocatalyst surface rich by ROSs and the pollutant molecules to be oxidized. Even though this option is the most effective, it received a lot of criticism because of the difficult handling of the suspension and recovery after the treatment which in turn leads to serious risks because of the toxicity of the nanoparticles (Haynes et al. 2017). Therefore, supporting of the photocatalyst on objects, e.g., glass or metallic plates, was a hot research topic since over more than two decades (Augugliaro et al. 2010, Djellabi and Ghorab 2015a, b). This approach would decrease the issue of nanoparticles toxicity; however, a significant decrease in the photocatalytic rates is usually obtained because of the low mass transfer (Bansal et al. 2016). Alternative solutions to bridge between the enhanced mass transfer and high photocatalytic rate have been suggested. One of them was the use of magnetic materials, wherein the photocatalyst is coated on magnetic iron (Djellabi et al. 2020, Bortolotto et al. 2022). The magnetic-based photocatalyst can be used as a suspension, keeping the highest photoactivity, and after the treatment, it can be recovered easily by applying an external magnetic field. One of the hottest topics in photocatalysis nowadays is the design of water floating photocatalysts (de Vidales et al. 2019, Djellabi et al. 2022a). This class of materials can be prepared by coating the photocatalysts on floating objects, or design self-floating photocatalysts (Djellabi et al. 2019b, Nasir et al. 2020). The advantages of floating photocatalysts are numerous as compared to other systems such as the maximum solar light absorption, high oxygenation rate, easy handling and recycling and can be used to oxidize floating pollutants, e.g., oily wastewaters. However, the design of highly stable floating material is still a serious issue. The coating of thin layer on floating objects would result in lower efficiency, and in addition, the release of photocatalyst particles is very common. Design of self-floating photocatalysts, even though a large quantity of photocatalyst is used, could be more effective and stable. Fully floating photocatalysts have the issue of mass transfer because only the pollutants at the top of water could interact with the photoactive material, while those in bulk water are excluded. The use of semi-floating photocatalysts that can float and move as well in the bulk water would be more interesting. In this work, TiO_2_ aerogel as a floating material obtained using of Borax, poly(vinyl alcohol) and polyvinylidene in the presence of H_2_O_2_ was designed. The photoactivity was tested towards the oxidation of three dyes and the photoreduction of Cr(VI) under solar light irradiation.

## Experimental

### Materials and chemicals

PVA (poly(vinyl alcohol)), 87–90% hydrolyzed, average mol wt 30,000–70,000, PVDF (polyvinylidene fluoride), average Mw ~ 534,000 and PVAC (poly(vinyl acetate)), average M_w_ ~ 500,000 were used to form the aerogel. Borax (Na_2_[B_4_O_5_(OH)_4_]0.8H_2_O). Borax anhydrous (≥ 99%) was used to make cross linking. Hydrogen peroxide solution, 30% (w/w) in H_2_O, was used as catalysis for aerogel synthesis. K_2_Cr_2_O_7_ (Merck, purity 99.9%) was used a source of Cr(VI). 1,5-Diphenylcarbazide (Merck, purity 98%) was utilized to make complexes with Cr(VI) for the analysis purpose. Tartaric acid (Fisher chemical, purity 100%) was used as hole scavenger in the photocatalytic Cr(VI) reduction experiments. Methyl orange (Sigma-Aldrich), rhodamine B (Sigma-Aldrich) and methyl red (Sigma-Aldrich) were used as dye pollutants. Titanium(IV) butoxide (Sigma-Aldrich, 97%) was used for the synthesis of TiO_2_.

### Synthesis and characterization of TiO_2_ aerogel

**Fig. 1:**
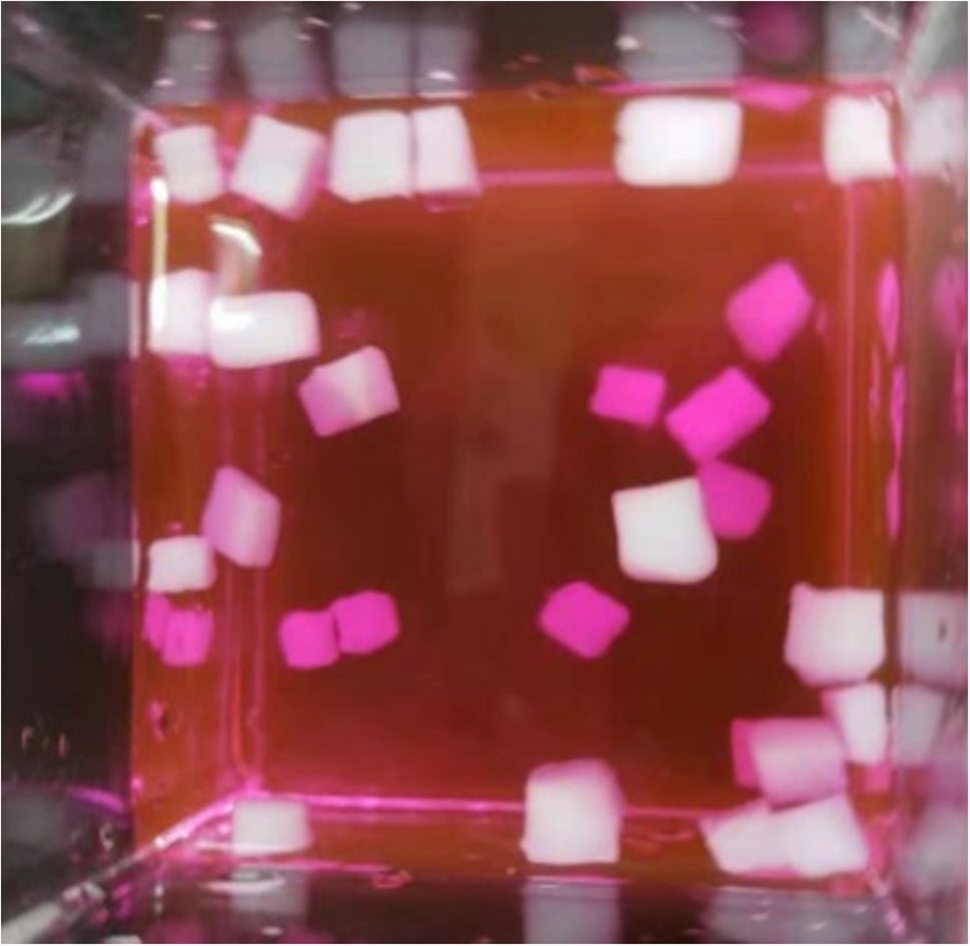
Photograph of TiO_2_ aerogel floating photocatalyst in RhB solution

Bare TiO_2_ was prepared by sol–gel method; 20 mL of Titanium(IV) n-butoxide was mixed in 40 mL of ethanol for 30 min, and 20 mL of distilled was added dropwise to the above solution and left under vigorous stirring for 2 h. The collected suspension was washed and dried at 100 °C overnight. After that, the suspension was calcined at 400 °C for 2 h at heating rate of 5 °C/min. TiO_2_ aerogel was prepared as follows: 100 mg of Na_2_[B_4_O_5_(OH)_4_]0.8H_2_O was stirred in 20 mL of hot distilled water for 30 min. After that, 0.5 g of PVA (poly(vinyl alcohol)) and 0.05 g of PVDF (polyvinylidene fluoride) were added to above solution; 1 mL of H_2_O_2_ (0.1 M) was added, and the solution was stirred for 1 h and sonicated for 30 min. Then, 0.2 g of TiO_2_ was added to the mixture and stirred vigorously for 2 h, followed by sonication for 30 min. PVAC (poly(vinyl acetate)) was added to form the gel, which then was washed several times with Na_2_[B_4_O_5_(OH)_4_]0.8H_2_O solution. The gel was then kept at − 20 °C overnight. After dried at room temperature, the solid gel was modelled as cubic form of few centimetres with mass weight of around 0.3 g for each. Figure 1 shows a photograph of TiO_2_ aerogel floating photocatalyst in RhB solution. TiO_2_ aerogel was characterized by XRD using a PANalytical X’PERT-PRO diffractometer. Diffused reflectance spectra of bare TiO_2_ and TiO_2_ aerogel were recorded on a Cary 5000 UV–Vis spectrophotometer (Agilent Technology).

### Photocatalytic experiments

The photocatalytic oxidation of dyes (methylene blue (MB), rhodamine blue (RhB) and methyl red (MR)) and reduction of Cr(VI) were carried out in a beaker exposed to solar light lamp irradiation. The concentration of dyes and Cr(VI) was 10 ppm. Before the irradiation of the solution, the photocatalyst and the solution were kept in contact for 30 min. Then, the light is turned on to carry out the photocatalytic oxidation/reduction of dyes or Cr(VI). In the case of Cr(VI), after dark adsorption, Cr(VI) was kept in contact with the photocatalyst for 30 min under light irradiation, and then tartaric acid was added as a hole scavenger. The concentration of dyes and Cr(VI) was followed by UV–Vis spectrophotometry. Cr(VI) was mixed with 1,5-diphenylcarbazide to form the complex before analysis.

## Results and discussion

**Fig. 2:**
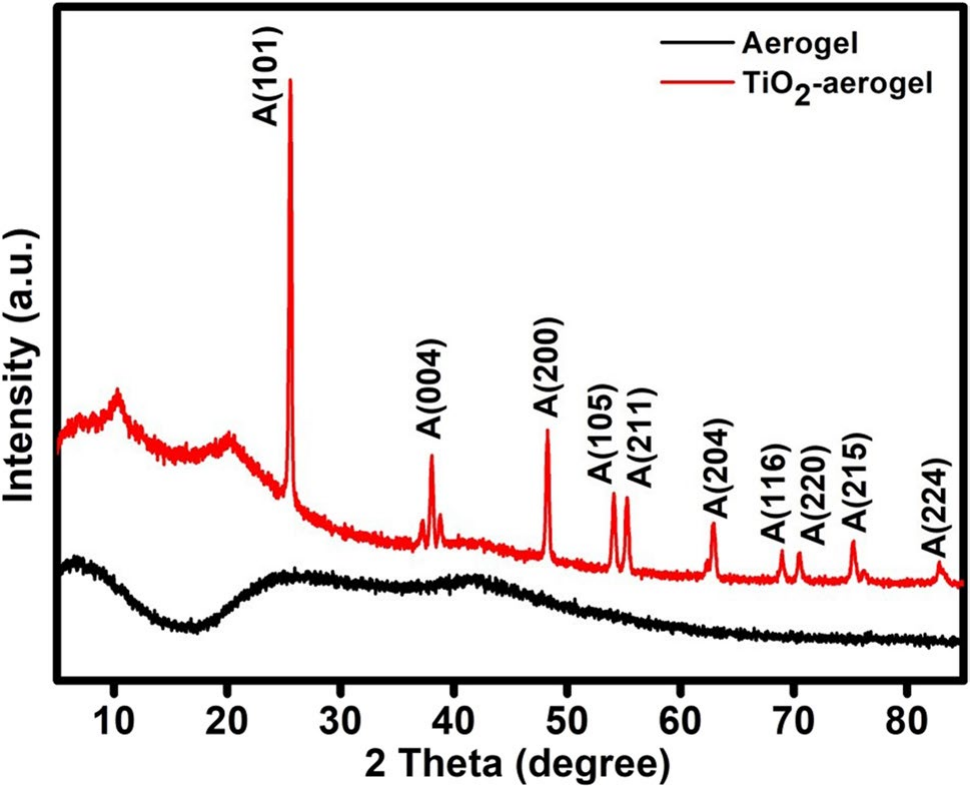
XRD spectra of bare TiO_2_ and TiO_2_ aerogel samples

**Fig. 3:**
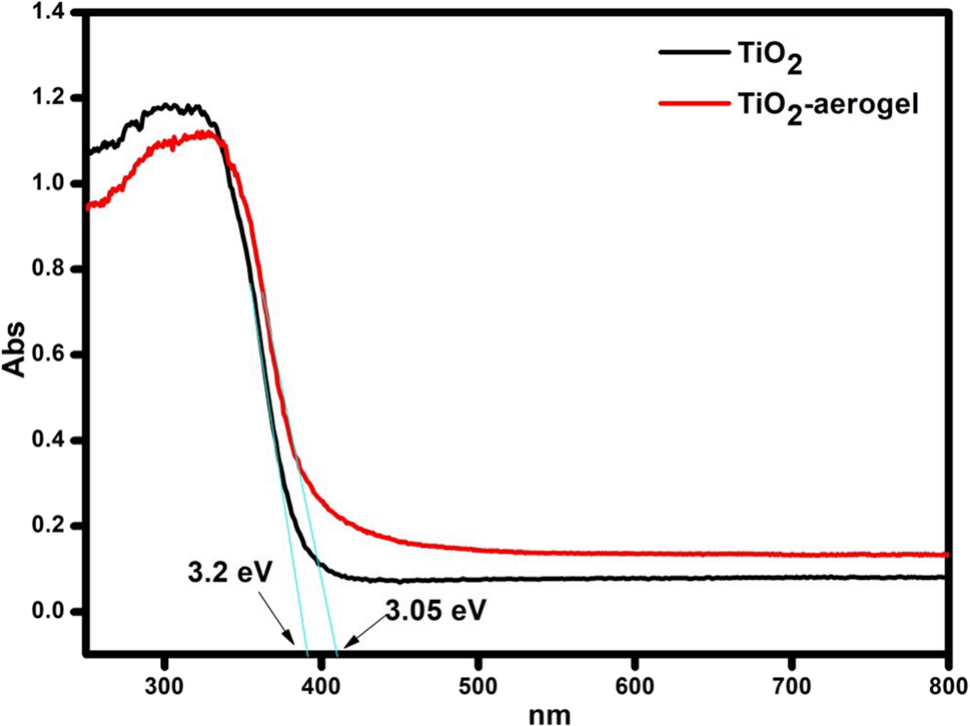
UV-DRS spectra of bare TiO_2_ and TiO_2_ aerogel samples

XRD patterns of crashed TiO_2_ aerogel and bare aerogel are shown in Fig. 2. Bare aerogel exhibits an amorphous structure without any specific indicative peaks. XRD spectrum of PVA usually shows a specific peak at around 2θ equal 19.1 (Chen et al. 2018); however, even though PVA content in the aerogel is high, its peak is not obvious in bare aerogel which is probably due its conjugation with PVDF and PVAC. In the case of TiO_2_ aerogel, intense characteristic peaks of anatase-TiO_2_ were produced which correspond to crystal planes of 101, 004, 200, 105, 211, 204, 116, 220, 215 and 224 (Djellabi et al. 2019a). Since the photocatalytic tests were carried out under solar light irradiation, the light response of TiO_2_ aerogel was checked via ultraviolet–visible diffuse reflectance spectroscopy (UV-DRS) analysis and the curves of bare TiO_2_ and TiO_2_ aerogel are shown in Fig. 3. It can be seen that there was a slight red shift in the light absorption of TiO_2_ aerogel as compared to bare TiO_2_. It might be due to the interaction of TiO_2_ with the aerogel compounds leads to form polymeric surface complexes. The evaluation of the direct band gap was performed by Kubelka–Munk equation, and the values were found to be 3.2 and 3.05 eV for TiO_2_ and TiO_2_ aerogel, respectively. This slight red shifting would help significantly the excitation of floating photocatalyst blocks.

**Fig. 4:**
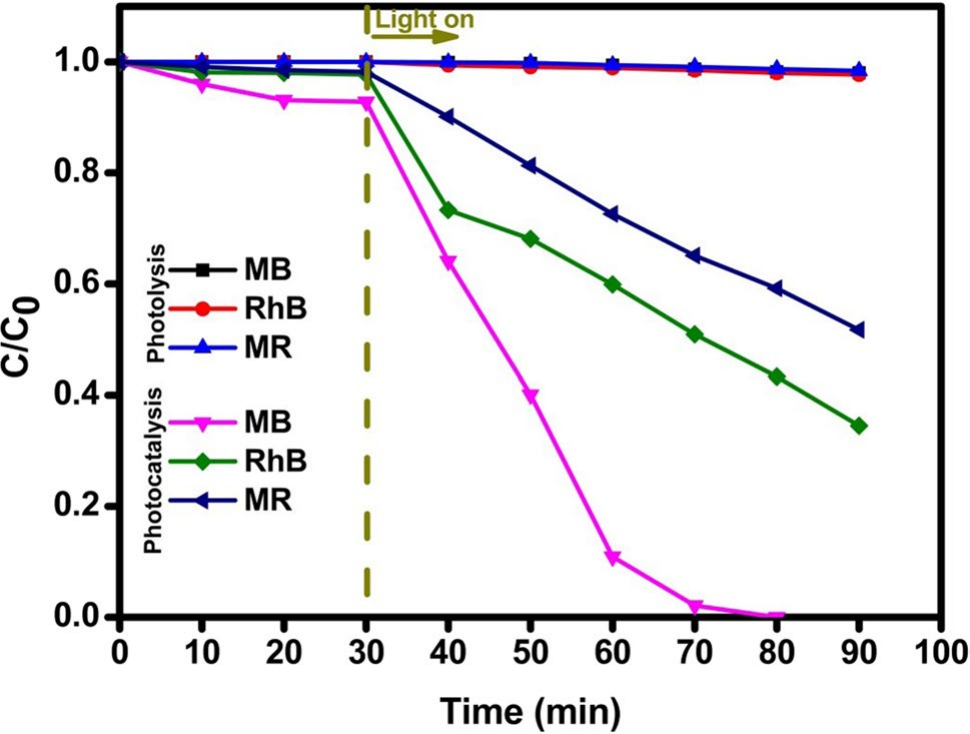
Photocatalytic activity of TiO_2_ aerogel towards the oxidation of dyes under solar light. [Dye]: 10 ppm

**Fig. 5:**
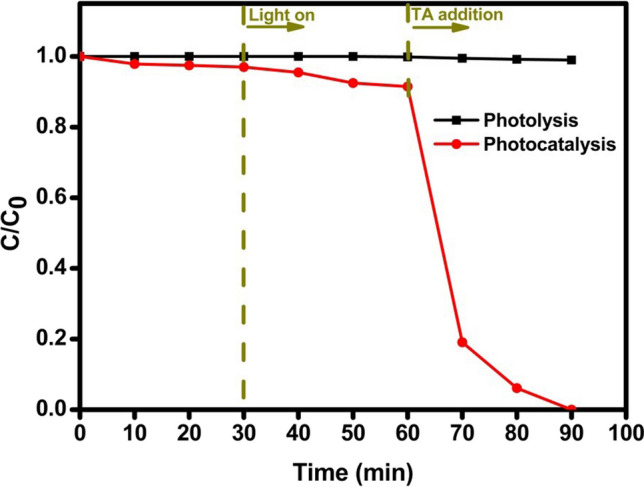
Photocatalytic reduction of Cr(VI) under solar light. [Cr(VI)]: 10 ppm, [Tartaric acid]: 10 ppm

The photocatalytic tests were carried out under solar light using TiO_2_ aerogel blocks towards the oxidation of three dyes (MB, RhB and MR) and the photoreduction of Cr(VI). The oxidation of dyes was performed at an initial concentration of 10 ppm and free pH (around 6.5) (Fig. 4), while the reduction of Cr(VI) was carried out at an initial concentration of 10 ppm and pH 3 with the addition of tartaric acid as a hole scavenger at a concentration of 10 ppm (Fig. 5). In dark, dyes and Cr(VI) do not show remarkable adsorption on the surface of TiO_2_ aerogel blocks because of their smooth surface. The oxidation of dyes by direct photolysis, as a control experiment, was negligible. Once the light is turned on in the presence of TiO_2_ aerogel, dyes have been subjected to oxidation by photoproduced ROSs. The photocatalytic oxidation rate of MB was the highest (total oxidation within 80 min). TiO_2_ aerogel could be active under solar light because of several facts. The solar spectrum contains around 5% of UV light which initiates the photoexcitation of TiO_2_. Even though the intensity of the light that is able to excite the photocatalyst is a very important parameter, however, a little energy is able to make TiO_2_ into action, especially at lower pollutant concentration (Fujishima et al. 2000). But most of recent reports proved that the photocatalytic rate is a light intensity dependency till a certain level where the light does not affect the photoexcitation process (Zang and Farnood 2008, Chong et al. 2010). The polymer-based aerogel can promote the visible absorption as a photosensitizer, wherein it can absorb light and transfer photoproduced charges to TiO_2_ (Wang et al. 2009, Riaz et al. 2015). In addition, as proved by UV-DRS analysis, a lower band gap was found in TiO_2_ aerogel, allowing enhanced photoexcitation under solar light. Coming to the different behaviour of photooxidation of dyes, it is important to point out that the ROS oxidation mechanism depends on many factors. The charge of dye molecule against the charge of the surface is very important as the first interaction (adsorption) is required to make the ROS oxidation reaction into action. ROSs mostly remain on the surface of the photocatalyst, and the better dye adsorption/interaction, the better ROSs oxidation would be obtained (Djellabi et al. 2014). Figure 4 shows that MB exhibits the best adsorption behaviour as compared to other dyes, but very slight. Indeed the adsorption of pollutants on the surface of the photocatalyst is very critical in photocatalytic oxidation; however, it is worth to mention that a huge adsorption of pollutant would not give an enhanced photooxidation as it might limit the penetration of light irradiation and the photoexcitation process (screen effect). This phenomenon occurs in single photocatalytic materials (naked photocatalyst); however, in the case of photoactive/adsorptive composites, i.e., TiO_2_-activated carbon (Abderrahim et al. 2022), BiPO_4_-Smectite (Fellah et al. 2022), CuO-Montmorillonite (Saber et al. 2021), the screen effect due of the high adsorption of pollutants is systematically reduced. In photoactive/adsorbing composites, Adsorb and Shuttle process takes place allowing the removal synergistically the pollutant from water. On the other hand, the redox reactions on the surface of the photocatalyst are potential dependency. The more the redox potential of dye molecule is negative than the VB potential level, the better direct oxidation by positive holes or ROSs produced on the VB is found. Thermodynamically, Gibbs energy change (ΔG), which is the potential gap between dye molecules, can be used to understand which molecule can be subjected to fast oxidation (Ohtani 2014). The photosensitizing effect of the dye itself can take place to promote the oxidation of dyes even at lower light energy as compared to the band gap of the photocatalyst. The adsorbed dye on the surface of the photocatalyst can absorb light and transfer photogenerated energy to the photocatalyst, resulting in dye destruction. The structure of the organic molecule is also a critical factor limiting the photocatalytic rate. Some organic molecules are easy to be oxidized because of the quick attack of ROSs, while others are more persistent.

In terms of Cr(VI) photoreduction (Fig. 5), the photogenerated electrons are transferred from the CB to Cr(VI) species to be reduced into Cr(III), known as direct reduction (Djellabi and Ghorab 2015a, b). Thermodynamically, the CB must be more negative than Cr(VI)/Cr(III) redox potential, and the more the gap between CB and Cr(VI)/Cr(III) potential is bigger, the more the photocatalytic reduction is effective. Indirect reduction occurs by some photoproduced such as ^−•^O_2_ (An et al. 2020, Hasija et al. 2021), which can reduce Cr(VI) into Cr(III). In direct photocatalytic reduction, there are two pathways: the first is slow reduction (only under light irradiation), and the second is very fast (under light and in the presence of hole scavenger). Without hole scavenger (only Cr(VI) in distilled water), the reduction of Cr(VI) by photogenerated is limited due to the recombination of charges and less accumulation of electrons on the conduction band. In this regard, the single electron transfer from the CB to Cr(VI) would take place, forming Cr(V) and Cr(IV) as intermediates, till total reduction to Cr(III) (Testa et al. 2001, Meichtry et al. 2007). However, the addition of hole scavenger molecule enhances the separation of electrons as it reacts with positive holes and oxidative ROSs, e.g., ^•^OH, avoiding the re-oxidation of photoproduced Cr(III) as discussed previously (Marinho et al. 2017a). In this case, the Cr(VI) would be reduced through one three-electron transfer process. In real wastewaters, organic pollutants and Cr(VI) often co-exist; therefore, probably the simultaneous oxidation of organic pollutants might act at the same time as a hole scavenging mechanism, and there is no need to add hole scavenger molecule.

**Fig. 6:**
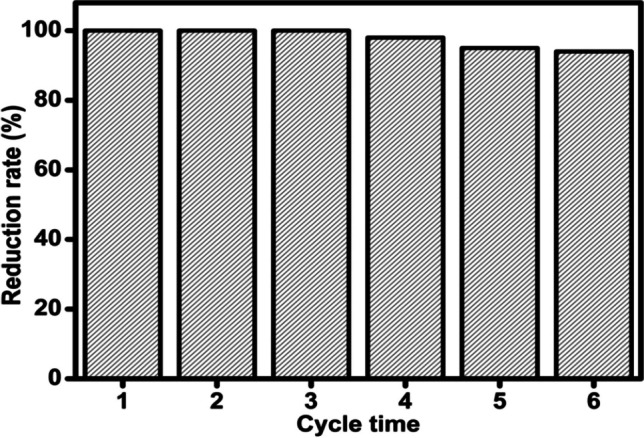
Recycling of TiO_2_ aerogel towards the reduction of Cr(VI) under solar light. [Cr(VI)]: 10 ppm, [Tartaric acid]: 10 ppm

The recycling of TiO_2_ aerogel was tested towards the Cr(VI) reduction under solar light several times (Fig. 6). After each cycle, TiO_2_ aerogel was washed and immersed for 20 min in borax solution to re-strengthen the texture of the aerogel, washed with distilled water and then dried. The tests were carried out similarly as shown in Fig. 4a. It was found out that TiO_2_ aerogel has significant physical and thermal stability to be used for several times. In fact, the immersion of TiO_2_ aerogel in borax solution can recover its physical texture.

The photocatalytic activity of TiO_2_ aerogel towards the reduction of Cr(VI) under solar light was compared with previous reported materials, and the results are listed in Table 1. It is important to mention that the comparison of the photoactivity of TiO_2_ aerogel with solar light responsive photocatalysts is not very accurate as TiO_2_ is not fully activated under solar light, and on top of that, the photocatalyst in the form of suspension would have higher surface area and photocatalytic activity because of the enhanced mass transfer. As above-mentioned, the objective of this work is to facilitate the real world application of photocatalytic technology via the use of semi-floating photocatalysts, rather than obtaining a highly effective photocatalyst for Cr(VI) reduction. We would like to mention that TiO_2_ can be replaced by any type of photocatalyst in this type of aerogel as reported in previous works (Djellabi et al. 2019b, 2021b). At the industrial level, not only the efficiency of the photocatalytic material is accounted by also other important factors such as easy handling, recycling, cost and sustainability.

**Table 1:** Recent reported studies on the photocatalytic reduction of Cr(VI)

Photocatalyst	[Cr(VI)]	Light	Reduction rate, time	Reference
Ni_3_S_2_-graphene	20 ppm	Visible light	90%, 180 min	(Hu et al. 2017)
Cu_2_In_2_ZnS_5_/Gd_2_O_2_S	80 ppm	Solar light	90%, 210 min	(Liu et al. 2019)
SnO_2_/SnS_2_/N-RGO	50 ppm	Visible light	100%, 230 min	(Wang et al. 2020)
Zr-SnS2/PANI	50 ppm	Visible light	100%, 220 min	(Zhang et al. 2022)
TCTA-PVP@Fe_3_O_4_	20 ppm	Solar light	80%, 60 min	(Djellabi et al. 2020)
Ca-Bi2O3	7 mM	Visible light	80%, 120 min	(Khosya et al. 2022)
Fe_3_O_4_/FeWO_4_	15 ppm	Visible light	100%, 220 min	(Ge et al. 2021)
Ag_3_PO_4_/Fe_3_O_4_	30 ppm	Solar light	90%, 120 min	(Bortolotto et al. 2022)
Ag/Ag_3_PO_4_@AC	50 ppm	Solar light	80%, 120 min	(Abderrahim et al. 2022)
TiO_2_ aerogel	10 ppm	Solar light	100%, 30 min	This work

**Fig. 7:**
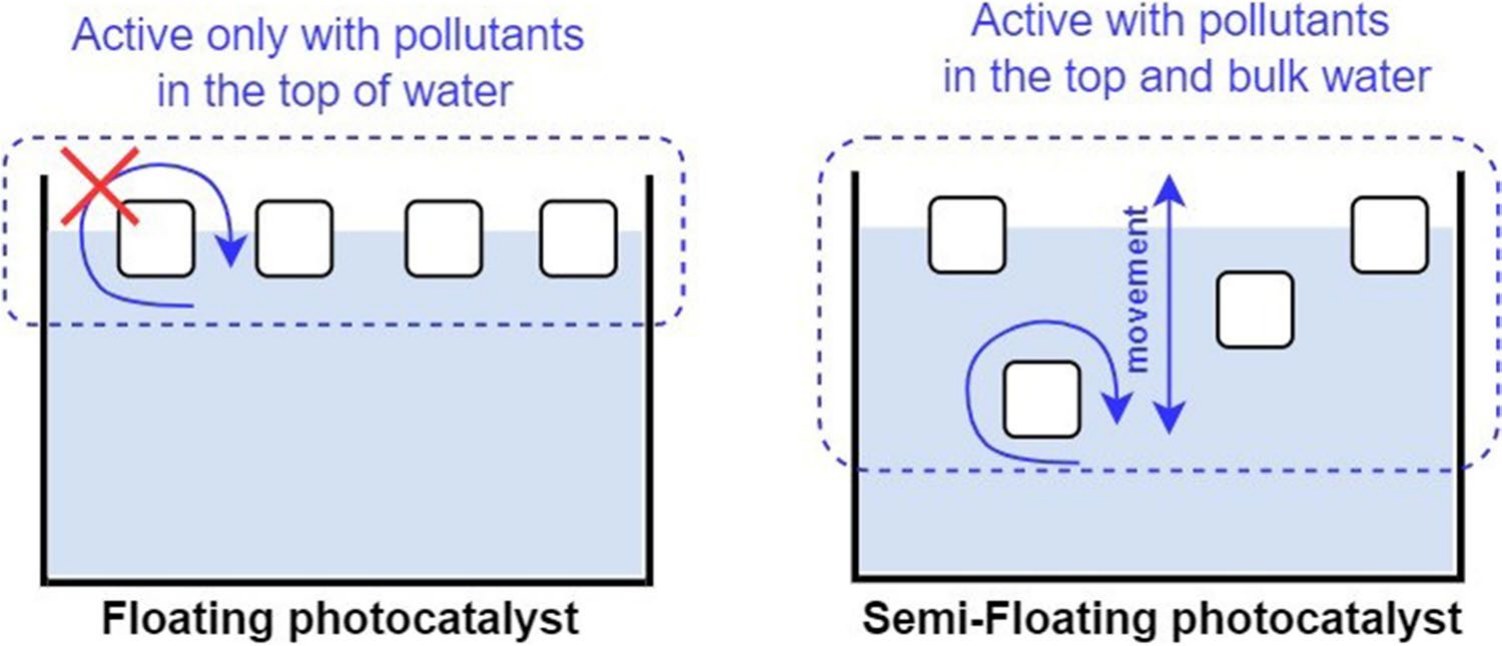
Behaviour of fully floating photocatalyst and semi-floating photocatalyst for solar photocatalytic water treatment

Compared to fully floating photocatalyst, TiO_2_ aerogel in this work showed a semi-floating behaviour wherein it can move from the top to the bulk deep solution. The concept of floating materials is to remain on the top of water and receive maximum oxygenation and light irradiation; therefore, a full photoexcitation and ROS generation could be obtained (Xing et al. 2018, Djellabi et al. 2021b). However, the design of floating materials in terms of shape is very critical. Transparent floating materials would allow the irradiation of all side of the photocatalyst making it more effective. Floating materials with shapes, e.g., balls, that allow them to move freely on the top of water can enhance the photoactivity and mass transfer and allow the use of all sides of photocatalyst as well. In some cases, floating materials show very weak photocatalytic rates because the top photoactive surface is not in touch with the pollutants in water. However, even though the design of floating photocatalyst is well shaped and designed, the capital drawback of floating materials is the low mass transfer in bulk solution. Pollutant species in deep water would not be in contact with the floating at large scale. On the contrary, semi-floating photocatalysts can move freely from the top to bulk water with a possible rotation, allowing an excellent mass transfer similar to that of suspension photocatalysts. Figure 7 summarizes the above discussed points, demonstrating the enhanced mass transfer in the case of semi-floating photocatalysts as compared to fully floating ones.

## Conclusions

In this work, TiO_2_ aerogel-based semi-floating material was designed, and its photoactivity was evaluated towards the oxidation of three dyes and photoreduction of Cr(VI) under solar light. The hybridization of TiO_2_ with the aerogel showed a slight red shift and a decrease in the band gap from 3.2 to 3.05 eV because of the fixation of polymer network on the surface of TiO_2_. The aerogel can act as a photosensitizer to initiate photocatalytic reaction even with wavelength slightly higher than 400 nm. The oxidation of dyes showed different photooxidation behaviours, wherein MB was the most subjected to ROS oxidation because of the better adsorption or/and better thermodynamic affinity with the valence band of TiO_2_. Cr(VI) photoreduction showed slower photoreduction rate without hole scavenger, while the addition of hole scavenger enhances significantly the photocatalytic rate which might be due to the enhanced one three-electron transfer process as a result of electrons accumulation on the conduction band. TiO_2_ aerogel could be recycled several times by using borax solution to recover the physical and texture of the TiO_2_ aerogel after each cycle. Semi-floating photocatalysts showed several technical advantages as compared to fully floating materials such as the high mass transfer in top and bulk water. This work shows some interesting insights in terms of solar floating photocatalysts for possible large-scale application.

## Data Availability

Not applicable.
